# Cell Surface Galectin-9 Expressing Th Cells Regulate Th17 and Foxp3^+^ Treg Development by Galectin-9 Secretion

**DOI:** 10.1371/journal.pone.0048574

**Published:** 2012-11-07

**Authors:** Souichi Oomizu, Tomohiro Arikawa, Toshiro Niki, Takeshi Kadowaki, Masaki Ueno, Nozomu Nishi, Akira Yamauchi, Toshio Hattori, Tsutomu Masaki, Mitsuomi Hirashima

**Affiliations:** 1 Department of Immunology and Immunopathology, Faculty of Medicine, Kagawa University, Kagawa, Japan; 2 Department of Biology, Kanazawa Medical University, Ishikawa, Japan; 3 GalPharma Co., Ltd., Kagawa, Japan; 4 Department of Holistic Immunology, Kagawa University, Kagawa, Japan; 5 Department of Inflammation Pathology, Faculty of Medicine, Kagawa University, Kagawa, Japan; 6 Life Science Research Center, Kagawa University, Kagawa, Japan; 7 Department of Breast Surgery, Kitano Hospital, Osaka, Japan; 8 Laboratory of Disaster-related Infectious Disease, International Research Institute of Disaster Science, Tohoku University, Sendai, Japan; 9 Department of Gastroenterology and Neurology, Faculty of Medicine, Kagawa University, Kagawa, Japan; Escola Paulista de Medicina - UNIFESP, Brazil

## Abstract

Galectin-9 (Gal-9), a β-galactoside binding mammalian lectin, regulates immune responses by reducing pro-inflammatory IL-17-producing Th cells (Th17) and increasing anti-inflammatory Foxp3^+^ regulatory T cells (Treg) *in vitro* and *in vivo*. These functions of Gal-9 are thought to be exerted by binding to receptor molecules on the cell surface. However, Gal-9 lacks a signal peptide for secretion and is predominantly located in the cytoplasm, which raises questions regarding how and which cells secrete Gal-9 *in vivo*. Since Gal-9 expression does not necessarily correlate with its secretion, Gal-9-secreting cells *in vivo* have been elusive. We report here that CD4 T cells expressing Gal-9 on the cell surface (Gal-9^+^ Th cells) secrete Gal-9 upon T cell receptor (TCR) stimulation, but other CD4 T cells do not, although they express an equivalent amount of intracellular Gal-9. Gal-9^+^ Th cells expressed interleukin (IL)-10 and transforming growth factor (TGF)-β but did not express Foxp3. In a co-culture experiment, Gal-9^+^ Th cells regulated Th17/Treg development in a manner similar to that by exogenous Gal-9, during which the regulation by Gal-9^+^ Th cells was shown to be sensitive to a Gal-9 antagonist but insensitive to IL-10 and TGF-β blockades. Further elucidation of Gal-9^+^ Th cells in humans indicates a conserved role of these cells through evolution and implies the possible utility of these cells for diagnosis or treatment of immunological diseases.

## Introduction

Galectin-9 (Gal-9) is a member of the galectin family of mammalian lectins and is characterized by its ability to bind β-galactoside. Gal-9 is expressed by the epithelium of the gastrointestinal tract, endothelial cells and several types of immune cells including T cells, B cells, macrophages and mast cells [1], [2]. Recently, the regulatory role of Gal-9 in excessive immunity has become evident. Gal-9 suppresses interleukin (IL)-17-producing effector T helper cells (Th)17 and Th1 [3]; these cells play an exacerbating role in the pathogenesis of various autoimmune diseases, whereas Gal-9 augments Foxp3^+^ regulatory T cells (Treg), an essential suppressor of excessive immunity [3]. In addition to increasing Treg, Gal-9 expands the population of monocytic myeloid-derived suppressor cells (MDSCs) [4], granulocytic MDSCs [5], [6], and plasmacytoid dendritic cell-like macrophages [7]–[9]. Induction of these regulatory cells appears to be a critical regulatory function of Gal-9.

The function of Gal-9 is thought to be exerted by binding to particular sets of carbohydrate moieties in receptor molecules expressed on the surface of target cells. Among several identified receptors of Gal-9, the T-cell immunoglobulin- and mucin-domain-containing molecule-3 (Tim-3) has been studied most extensively. Binding of Gal-9 to Tim-3 expressed by activated Th1 and/or Th17 triggers cellular apoptosis and terminates Th1/Th17-skewed immunity [10], [11]. Gal-9 must be secreted by some types of cells to initiate this response. However, Gal-9 lacks a signal sequence essential for secretion via the canonical endoplasmic reticulum (ER)-Golgi pathway and is located predominantly in the cytoplasm where it plays roles in protein sorting and transcriptional regulation of cytokine genes [12], [13]. Gal-9 secretion has been demonstrated in several cell lines [14], [15], but the mechanism of Gal-9 translocation through the lipid bilayer, as well as the identification of Gal-9-secreting cells *in vivo* has yet to be elucidated. Gal-9 expression and secretion are not always correlated. Recently, Wang *et. al*. suggested through indirect observation that Treg secretes Gal-9 and ameliorates Th1 responses [16]. From a functional perspective of the protein, Gal-9 may be secreted by regulatory cells, including Treg.

We hypothesize that Gal-9-secreting cells might express Gal-9 on the cell surface as a translocation intermediate and may be identified by staining the cells with specific antibodies. The goal of this study was to identify Gal-9-secreting cells. We identified CD4 T cells expressing Gal-9 on their surfaces (Gal-9^+^ Th cells). Gal-9^+^ Th cells secrete Gal-9 upon T cell receptor (TCR) stimulation, but other CD4 T cells lacking Gal-9 on the surface do not, although they express indistinguishable amounts of Gal-9 intracellularly. The characteristics of Gal-9^+^ Th cells and the significance of these cells in immunoregulation are discussed.

## Results

### Gal-9-secreting Cells Emerge from Naïve CD4 T cells by TCR Stimulation

**Figure 1:**
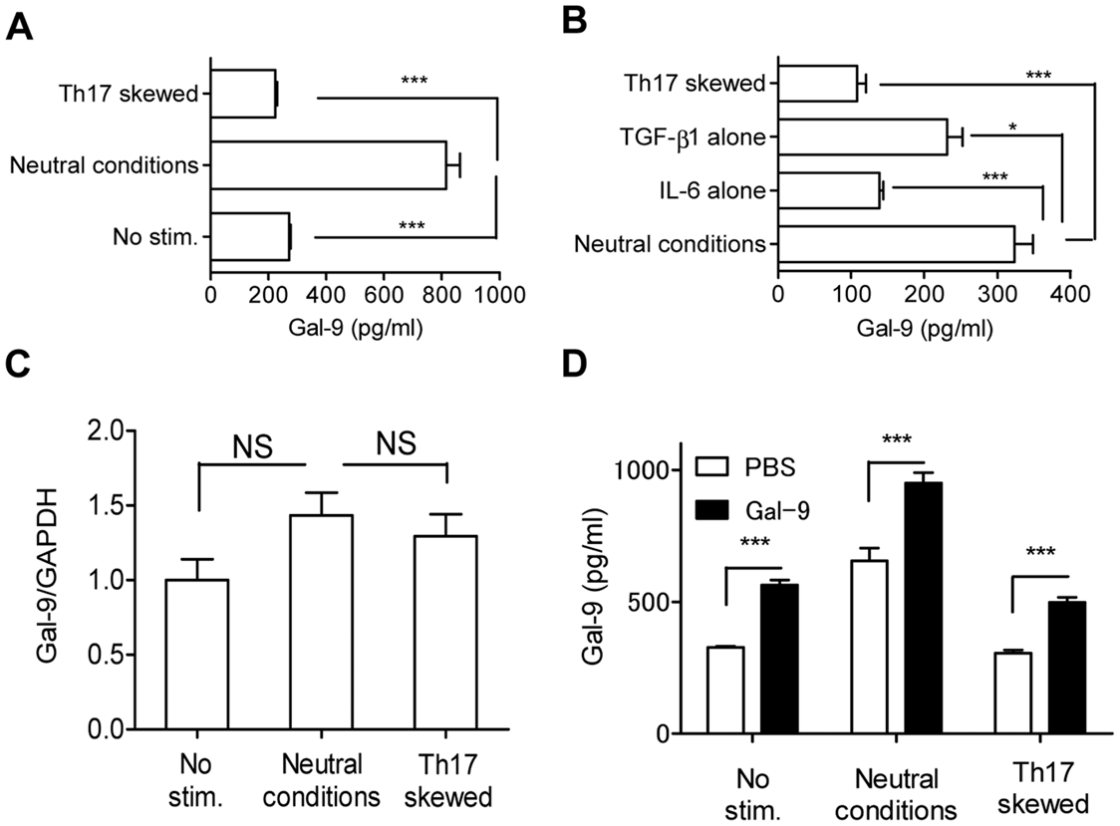
Gal-9 secretion in naïve CD4 T cell culture upon TCR stimulation. (**A**) Naïve CD4 T cells were cultured in Th17-skewing conditions, neutral conditions (TCR stimulation), or without stimulation for 96 h before released Gal-9 was measured using ELISA. (**B**) Same as (**A**), except that the cells were cultured under Th17-skewing conditions, neutral conditions, neutral conditions plus TGF-β1 (TGF-β1 alone), or neutral conditions plus IL-6 (IL-6 alone). (**C**) Gal-9 mRNA was quantified using real-time RT-PCR. (**D**) Gal-9 secretion in the presence of human stable Gal-9 (30 nM) was measured. The human protein was not detected by mouse Gal-9 ELISA (see text for details). All results are shown as the mean ± SEM of quadruplicates. *p*<0.001 (***), *p*<0.05 (*), not significant (NS). Representative data out of at least 2 experiments are shown.

We recently found that exogenous Gal-9 suppresses Th17 development and simultaneously enhances Treg development *in vitro*, even under Th17-skewing conditions, in an IL-2-dependent but Tim-3-independent manner [11]. These results suggest that endogenous Gal-9 plays a role in Th17/Treg development and that Gal-9 production and/or secretion may be suppressed under Th17-skewing conditions. To confirm this, we examined Gal-9 secretion from naïve CD4 T cells cultured under neutral and Th17-skewing conditions. As expected, Gal-9 was secreted under neutral conditions, whereas secretion was suppressed under Th17-skewing conditions (**Figure 1A**) largely because of the presence of IL-6 in the culture (**Figure 1B**). Despite differences in secretion capability, Gal-9 mRNA expression level did not differ between neutral and Th17-skewing conditions (**Figure 1C**). We further examined whether exogenous Gal-9 stimulates endogenous Gal-9 secretion. For this experiment, 30 nM of recombinant human stable Gal-9 was added to the culture, and the secretion of mouse Gal-9 was monitored using a specific enzyme-linked immunosorbent assay (ELISA) for mouse Gal-9. Human recombinant Gal-9 cross-reacts with mouse cells biologically and has been used in various rodent studies, but it was not detected in our mouse Gal-9 ELISA (**Figure S1**). As seen in **Figure 1D**, exogenous Gal-9 enhanced Gal-9 secretion even under Th17-skewing conditions. These results suggest that (1) Gal-9-secreting cells are present under neutral conditions, but the secretion capability and/or the number of Gal-9-secreting cells is reduced under Th17-skewing conditions, largely by IL-6, and (2) exogenous Gal-9 counteracts the inhibition of Gal-9-secretion even under Th17-skewing conditions.

### Surface Gal-9-expressing Th cells Secrete Gal-9

We hypothesized that Gal-9 could be detected on the surface of Gal-9-secreting cells as an intermediate during translocation through the lipid bilayer. We thus performed flow cytometry to measure surface Gal-9 expression using cells stimulated as described in **Figure 1A**. During secretion, Gal-9 likely binds to adjacent cells via their cell surface glycoproteins or glycolipids, which may complicate the identification of Gal-9-secreting cells. Therefore, Gal-9 staining was performed in the presence of 30 mM lactose, because this concentration of lactose is sufficient for removing exogenously added Gal-9 bound on the cell surface without affecting Gal-9 staining (**Figure S2A**).

**Figure 2:**
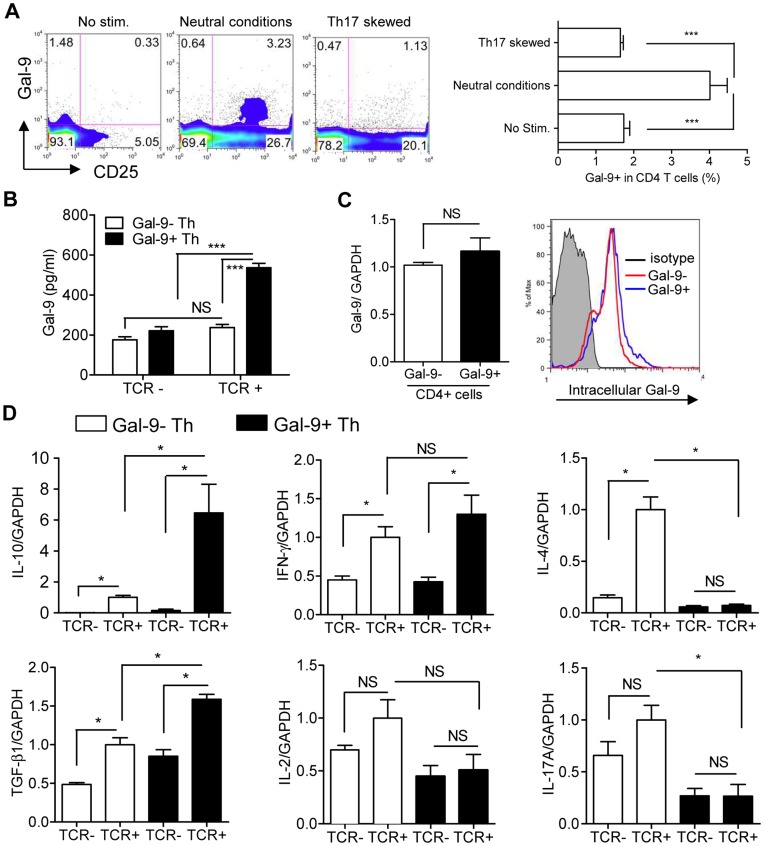
Identification of Gal-9^+^ Th cells. (**A**) Naïve CD4 T cells were cultured as in **Figure 1A**, and cell-surface Gal-9 expression was monitored using flow cytometry. (**B**) Naïve CD4 T cells were sorted into Gal-9^+^ Th and Gal-9^−^ Th cells with a cell sorter and cultured under TCR stimulation or left unstimulated for 4 days before Gal-9 secretion into the culture media was measured. (**C**) Gal-9^+^ Th and Gal-9^−^ Th cells cultured under TCR stimulation for 4 days were examined for Gal-9 mRNA expression (left) and intracellular Gal-9 protein expression (right). (**D**) Cytokine mRNA expression in Gal-9^+^ Th and Gal-9^−^ Th cells cultured with or without TCR stimulation for 4 days. All the results are shown as the mean ± SEM of quadruplicates. *p*<0.001 (***), *p*<0.05 (*), not significant (NS). Representative data out of at least 2 experiments are shown.

Gal-9 staining revealed the existence of surface Gal-9-expressing CD4 T cells (Gal-9^+^ Th cells). The frequency of Gal-9^+^ Th cells was approximately 1.5% without TCR stimulation and was increased to approximately 4% after TCR stimulation under neutral conditions (**Figure 2A**). Interestingly, the frequency of Gal-9^+^ Th cells as well as CD25^+^ CD4 T cells was reduced under Th17-skewing conditions (**Figure 2A**). Surface Gal-9 stably adhered to the cell surface during staining at 4°C even in 100 mM lactose (**Figure S2B**). Gal-9 secretion (**Figure 1A**) correlates well with the emergence of Gal-9^+^ Th cells (**Figure 2A**). To confirm our hypothesis that these Gal-9^+^ Th cells are the primary source of secreted Gal-9, Gal-9^+^ and Gal-9^−^ naïve Th cells were isolated using a cell sorter (**Figure S3**), cultured under neutral conditions and examined for Gal-9 secretion by ELISA. Consistent with our hypothesis, Gal-9^+^ Th cells, but not Gal-9^−^ Th cells, secreted Gal-9 upon TCR stimulation (**Figure 2B**). This is the first report of identification of Gal-9-secreting Th cells and demonstrates a useful technique for detecting cells with Gal-9 secretion capability. Enigmatically, expression levels of Gal-9 mRNA and intracellular Gal-9 protein did not show any apparent differences between Gal-9^+^ and Gal-9^−^ Th cells stimulated under neutral conditions (**Figure 2C**). Cytokine mRNA measurement demonstrated that compared to Gal-9^−^ Th cells Gal-9^+^ Th cells induced significantly higher levels of IL-10 and TGF-β upon TCR stimulation (**Figure 2D**). Induction of IFN-γ was similar to Gal-9^−^ Th cells, while IL-2, IL-4 and IL-17 were not induced by Gal-9^+^ Th cells (**Figure 2D**).

The frequency of Gal-9^+^ CD25^−^ Th cells in various lymphoid organs of normal mice was determined using flow cytometry. Approximately 25% of CD4 single-positive cells in thymus cells expressed surface Gal-9. Among CD25^−^ CD4 T cells, approximately 4% in lymph nodes, 7% in spleen and peripheral blood mononuclear cells and 15% in Peyer’s patches expressed surface Gal-9 (**Table 1** **and Figure S5**).

**Table 1:** Frequency of Gal-9^+^ Th cells in various organs in mice.

Organs	Phenotype	% in Gal-9^+^ CD25^−^ cells
		Mean ± SD
Thymus	in CD4 SP T cells	24.6±0.4
LN	in CD4 T cells	3.6±0.4
Spleen	in CD4 T cells	6.5±0.8
Peyer’s patches	in CD4 T cells	14.8±0.7
PBMC	in CD4 T cells	7.2±0.9

Lymphocytes from indicated organs (n = 3) were stained with anti-CD3, anti-CD4, anti-CD25, and anti-Gal-9 antibodies and analyzed using flow cytometry. SP: single positive, LN: lymph node.

### Gal-9^+^ Th cells are Different from Treg

**Figure 3:**
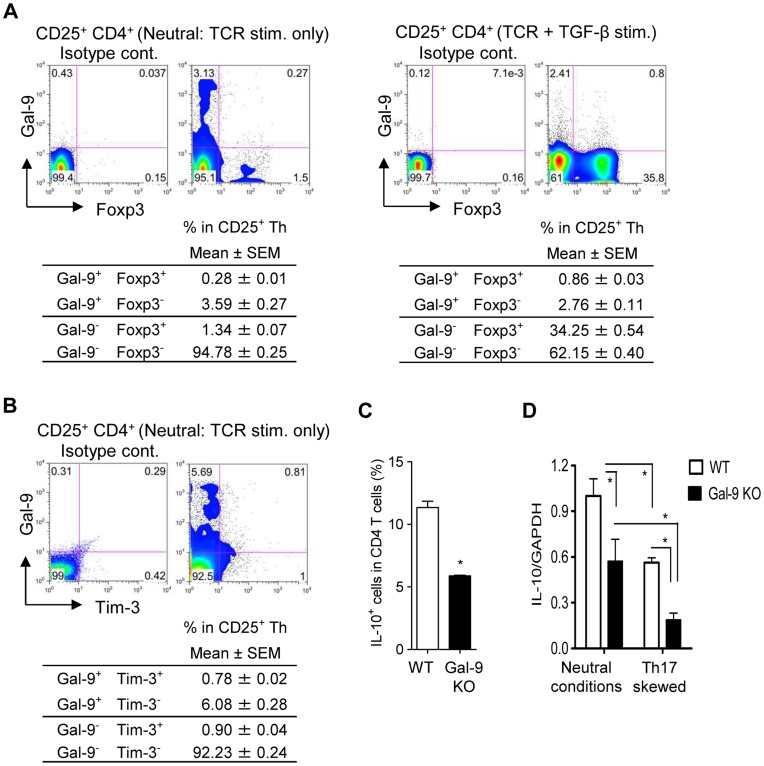
Phenotype of Gal-9^+^ Th cells. To clearly demonstrate the co-expression of cell-surface Gal-9 with either Foxp3 or Tim-3, highly sensitive Gal-9 staining with biotinylated-anti-Gal-9 antibody plus streptavidin-APC was employed. (**A**) Naïve CD4 T cells cultured under neutral conditions (left) or Treg-skewing conditions (right) for 4 days were examined for Foxp3 (Treg marker) and cell-surface Gal-9 expressions using flow cytometry. (**B**) Co-expression analysis of cell-surface Gal-9 and Tim-3 in naïve CD4 T cells cultured under neutral conditions for 4 days. (**C**) Intracellular IL-10 in naïve CD4 T cells purified from wild-type (WT) or Gal-9 knockout (Gal-9 KO) mice. (**D**) Naïve CD4 T cells from WT and Gal-9 KO mice were cultured under neutral or Th17-skewing conditions, and IL-10 mRNA expression was measured. Results are shown as the mean ± SEM of triplicate or quadruplicate. Symbol (*) represents significant (*p*<0.05) differences from the indicated counterparts. Representative data out of at least 2 experiments are shown.

Recently, Treg was suggested to secrete Gal-9 to suppress Th1 immunity [16]. As shown in **Figure 2D**, the expression of IL-10 and TGF-β by Gal-9^+^ Th cells appears to further support the identity between Treg and Gal-9^+^ Th cells. However, Gal-9^+^ Th cells were devoid of Foxp3 expression while Foxp3^+^ Th cells were devoid of surface Gal-9 expression (**Figure 3A**). Therefore, Gal-9^+^ Th cells were clearly a different population from Foxp3^+^ Treg and rarely co-expressed Tim-3 (**Figure 3B**). In Gal-9 knockout mice, the frequency of IL-10^+^ cells was significantly lower in CD4 T cells compared to those from wild type mice (**Figure 3C**). As demonstrated in **Figure 2A**, the number of Gal-9^+^ Th cells increased under neutral conditions but decreased under Th17-skewing conditions. Similarly, IL-10 expression decreased under Th17-skewing conditions and was further decreased by Gal-9 deficiency (**Figure 3D**). These observations suggest a close relationship between Gal-9^+^ Th cells and IL-10-producing CD4 T cells.

### Gal-9+ Th cells Regulates Th17/Treg Development by Gal-9

**Figure 4:**
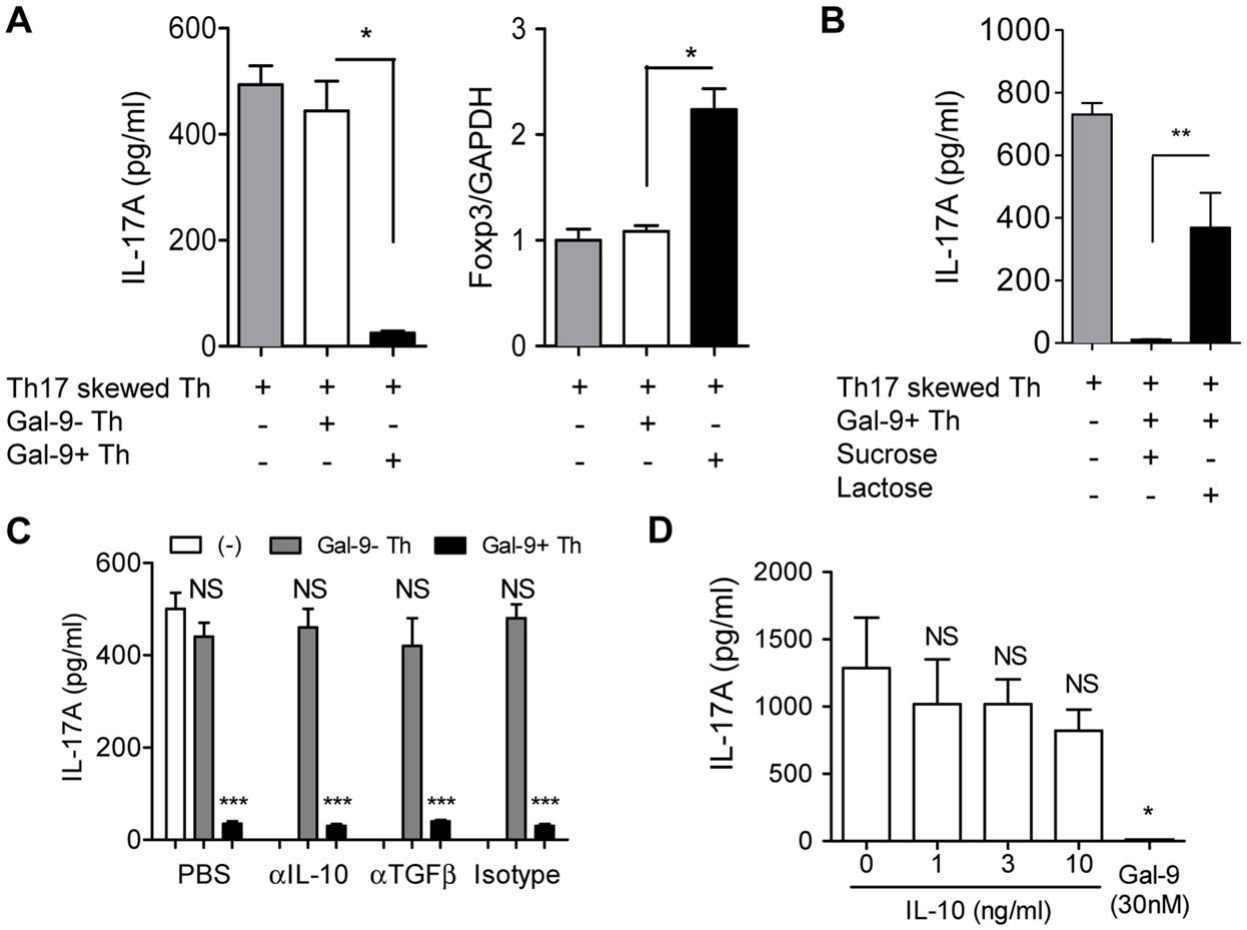
Regulatory function of Gal-9^+^ Th cells on Th17/Treg development. (**A–C**) Gal-9^+^ Th and Gal-9^−^ Th cells were obtained using a cell sorter from naïve CD4 T cells cultured under neutral conditions for 4 days. These cells were co-cultured with Th17-skewed cells at 1∶1 ratio and cultured for 90 h before analysis. (**A**) IL-17A and Foxp3 expression measured using ELISA and real-time RT-PCR, respectively. (**B**) IL-17A production in the Gal-9^+^ Th cell co-culture was examined in the presence of Gal-9 antagonist lactose or irrelevant sugar sucrose. (**C**) IL-17A production was examined in the presence of anti-IL-10 or anti-TGF-β blocking antibodies. (**D**) Naïve CD4 T cells were cultured under Th17-skewing conditions for 4 days in the presence of indicated concentration of IL-10 or human stable Gal-9 before IL-17A was measured. Results are shown as the mean ± SEM of quadruplicate experiments. *p*<0.001 (***), *p*<0.01 (**), *p*<0.05 (*), not significant (NS). Representative data out of at least 2 experiments are shown.

When naïve CD4 T cells committed to Th17 development were co-cultured with Gal-9^+^ Th cells (1∶1), IL-17A production was significantly suppressed, whereas Foxp3 expression was reciprocally induced (**Figure 4A**). Suppression of IL-17A production by Gal-9^+^ Th cells was abrogated by lactose, an antagonist of Gal-9, but not by sucrose (**Figure 4B**). The induction and function of Th17 are known to be regulated by cytokines secreted by other major T cell subsets, including IFN-γ, IL-4, IL-10, and TGF-β [17]. Since IL-10 and TGF-β are highly expressed by Gal-9^+^ Th cells, we examined the effect of blocking antibodies against IL-10 and TGF-β. However, these cytokine blockades did not affect the regulatory activity of Gal-9^+^ Th cells (**Figure 4C**). Furthermore, the addition of recombinant IL-10 did not suppress IL-17A production in our assay system (**Figure 4D**).

### Expansion of Gal-9^+^ Th cells by Exogenous Gal-9

**Figure 5:**
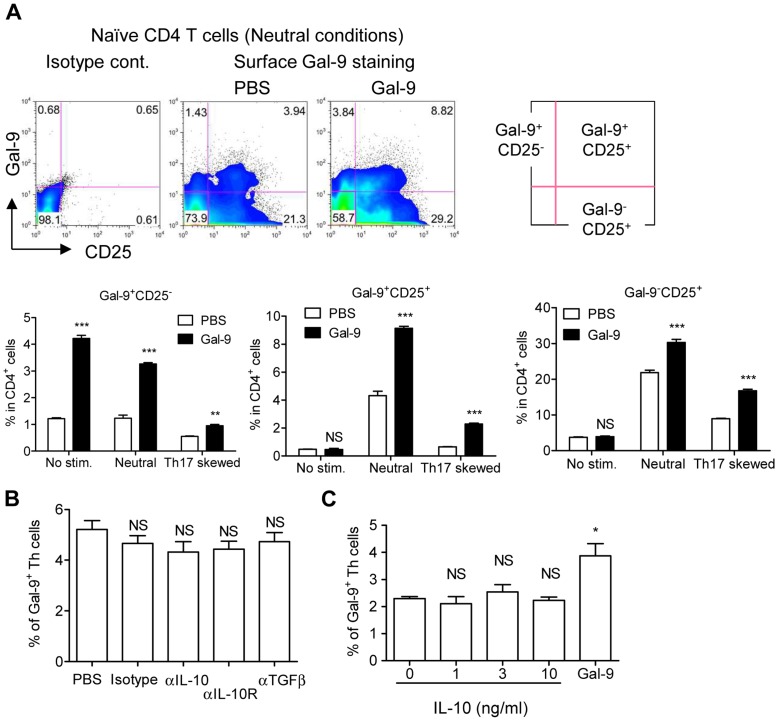
Expansion of Gal-9^+^ Th cells by exogenous Gal-9. (**A**) Naïve CD4 T cells were cultured under unstimulated, neutral, or Th17-skewing conditions for 4 days in the presence or absence of 30 nM human stable Gal-9 before surface Gal-9 expression was monitored by flow cytometry using an anti-mouse Gal-9 antibody. The antibody does not cross-react with the added human Gal-9. Dot plots are representative results obtained from neutral conditions in the presence or absence of exogenous Gal-9. (**B and C**) Flow cytometric analysis of Gal-9^+^ Th cell frequency after 4-day culture of naïve CD4 T cells under neutral conditions in the presence of blocking anti-IL-10 or anti-TGF-β antibody (**B**) or in the presence of the indicated concentration of IL-10 or 30 nM human stable Gal-9 (**C**). Results are means ± SEMs of quadruplicates. *p*<0.001 (***), *p*<0.01 (**), *p*<0.05 (*), not significant (NS). Representative data out of 2 experiments are shown.

As demonstrated in **Figure 1D**, the addition of recombinant Gal-9 to naïve CD4 T cell cultures augmented Gal-9 secretion. This observation indicates that exogenous Gal-9 either activates or increases Gal-9^+^ Th cells (or both). To address the question, naïve CD4 T cells were cultured for 4 days with no stimulation, or under neutral- or Th17-skewing conditions in the presence or absence of recombinant human stable Gal-9, and the frequency of Gal-9^+^ Th cells was monitored using flow cytometry. As shown in **Figure S1**, our anti-mouse Gal-9 antibody did not cross-react with the recombinant human protein and could detect Gal-9^+^ Th cells. Exogenous Gal-9 increased the numbers of Gal-9^+^ CD25^−^ Th cells irrespective of TCR stimulation (**Figure 5A**). Upon TCR stimulation, exogenous Gal-9 increased the numbers of both Gal-9^+^ CD25^+^ and Gal-9^−^ CD25^+^ Th cells under neutral and Th17-skewing conditions (**Figure 5A**).

Two major cytokines expressed by Gal-9^+^ Th cells, IL-10 and TGF-β, were tested to determine whether they affect the expansion of Gal-9^+^ Th cells. Blocking antibodies specific against IL-10 and TGF-β added to naïve CD4 T cell cultures under neutral conditions for 4 days did not affect the number of Gal-9^+^ Th cells (**Figure 5B**). IL-10 has been shown to expand type-1 regulatory T cells (Tr1), which are known to strongly express IL-10 and may therefore be related to Gal-9^+^ Th cells [18]–[20]. However, recombinant IL-10 at 10 ng/mL did not increase Gal-9^+^ Th cells in 4-day cultures, whereas exogenous Gal-9 increased the number of cells (**Figure 5C**).

### Gal-9^+^ Th cells in Humans

**Figure 6:**
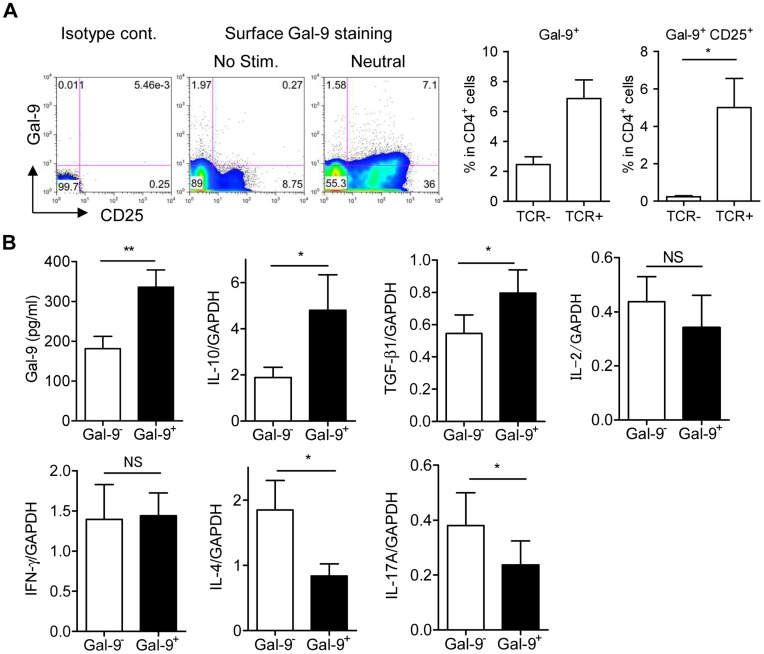
Gal-9^+^ Th cells in humans. (**A**) CD4 T cells from human peripheral blood were cultured with or without TCR stimulation for 4 days. Surface Gal-9 and CD25 expression was analyzed using flow cytometry. Results are shown as the mean ± SEM from 4 healthy donors. (**B**) CD4 T cells were cultured under neutral conditions for 4 days and then sorted into Gal-9^+^ CD25^+^ Th cells and Gal-9^−^ CD25^+^ Th cells. Sorted cells were cultured under neutral conditions for 4 days before the measurement of secreted Gal-9 by ELISA or cytokine mRNA using real-time RT-PCR. Results are shown as the mean ± SEM from 8 healthy donors. * *p*<0.05. Data from 2 representative experiments are shown.

Recombinant Gal-9 functions in human peripheral blood CD4 T cells to augment Foxp3^+^ Treg development while suppressing Th17 development (**Figure S4**). These observations demonstrate that the function of Gal-9 in terms of Th17/Treg development is equivalent between humans and mice, and implies the existence of Gal-9^+^ Th cells in humans. We examined peripheral CD4 T cells from normal subjects to determine whether a surface Gal-9-expressing population could be observed. In accordance with the findings in mouse studies, flow cytometric analysis revealed the presence of Gal-9^+^ Th cells in peripheral blood mononuclear cells (PBMC) and the expansion of the cells by TCR stimulation (**Figure 6A**). To examine the characteristics of human Gal-9^+^ Th cells, peripheral CD4 T cells were cultured under neutral conditions for 4 days to allow the expansion of Gal-9^+^ Th cells, were sorted into Gal-9^+^ and Gal-9^−^ Th cells according to surface Gal-9 expression, and were then cultured for another 4 days under neutral conditions before analysis. Human Gal-9^+^ Th cells secreted higher amounts of Gal-9 and expressed higher levels of IL-10 and TGF-β mRNA compared to Gal-9^−^ Th cells. Expression of IL-2 and IFN-γ did not differ between the 2 populations, whereas the levels of IL-4 and IL-17 were significantly lower in Gal-9^+^ Th cells (**Figure 6B**). These characteristics of human Gal-9^+^ Th cells are identical to those observed in mouse Gal-9^+^ Th cells (**Figure 2**).

## Discussion

Gal-9 has been demonstrated to suppress hyper immune reactions through several modes of action, including the induction of apoptosis in Tim-3^+^ Th1 and Th17, and suppression of Th17 development with concomitant induction of Treg [3], [10], [11]. These regulatory functions of Gal-9 have been elucidated primarily by pharmacological studies *in vitro* and *in vivo* during which recombinant Gal-9 was administered. However, the cells responsible for Gal-9 secretion have not been resolved. Here, we identified Gal-9^+^ Th cells that express Gal-9 on their surfaces secrete Gal-9 upon TCR stimulation, and regulate Th17/Treg development. Recently, Treg was reported to secrete Gal-9 and suppress Th1. We clarified that Gal-9^+^ Th cells are devoid of Foxp3 expression and are the predominant CD4 T cells secreting Gal-9. The discovery of Gal-9^+^ Th cells will improve the understanding of complex immunoregulation by Gal-9, provide a tool to study expression control and secretion mechanisms of Gal-9, and may provide clinical utilities for diagnosis or cell-based therapy in the future.

It is known that the administration of recombinant Gal-9 modulates immunity bidirectionally, not only suppressing excessive immunity and inflammation but also enhancing these functions in the context of compromised immunity [21], [22]. It is not clear whether Gal-9^+^ Th cells boost immunity or further suppresses it under hypo immune conditions; this can be examined using animal models such as tumor-bearing mice.

We showed that Gal-9 derived from Gal-9^+^ Th cells play a vital role in regulating Th17/Treg development because the effect of Gal-9^+^ Th cells was abrogated by a Gal-9 antagonist. However, the concentration of Gal-9 secreted into the culture media was 500–800 pg/mL, which was significantly lower than the effective concentration for recombinant Gal-9 to elicit the same effect (30 nM: approximately 1 µg/mL). We hypothesize that Gal-9^+^ Th cells secrete Gal-9 in close proximity to target cells via a paracrine mechanism that may involve cell-cell contact to identify and confirm the target cells and achieve efficient and secure regulation by Gal-9. It cannot be ruled out that cell-surface Gal-9 may also be involved for the regulation by directly interacting with target molecules.

Contrary to expectation, both Gal-9^+^ Th cells and other CD4 T cells express Gal-9 in the cytoplasm at comparable levels. Therefore, it is plausible that Gal-9^+^ Th cells possess secretion machinery that is absent in Gal-9^−^ Th cells. Gal-9, like other galectins, does not contain a signal sequence, and the secretion mechanism has remained obscure. In one study examining Gal-1, secretion was found to require a counter-receptor, and translocation through the plasma membrane was found to be energy-independent [23]. In this case, a membrane pore must be present to enable Gal-1 translocation. Conversely, Gal-9 is secreted as a component of the exosome in the case of Epstein-Barr virus–infected nasopharyngeal carcinoma [24]. Whether this mechanism is true for Gal-9 secretion from Gal-9^+^ Th cells or whether there is a third mechanism remains to be determined.

Using human peripheral T cells, we demonstrated that Gal-9^+^ Th cells are present in humans, and the immunoregulatory function of Gal-9 on Th17/Treg appears to be equivalent between humans and mice. These findings suggest clinical applications of recombinant Gal-9 and Gal-9^+^ Th cells. We found that IL-6 abrogates the increase of Gal-9^+^ Th cells *in vitro*. Thus, neutralization of IL-6 may be a strategy for increasing Gal-9^+^ Th cells in order to ameliorate Th1/Th17-skewed immunity. An anti-IL-6 receptor-neutralizing antibody, tocilizumab, has been used to treat rheumatoid arthritis. Currently, the primary mechanism is explained by the suppression of Th17 cell development by IL-6 [25]; however, an additional cryptic mechanism of the antibody may involve efficient induction of Gal-9^+^ Th cells. It would be interesting to measure Gal-9^+^ Th cells in patients receiving IL-6 blockades, because this may demonstrate the utility of Gal-9^+^ Th cells as surrogate markers to judge the effectiveness of the therapy.

Gal-9^+^ Th cells must be further characterized to assess clinical utilities, including cell-based therapies as have been attempted for Tr1 and Treg, for the treatment of refractory autoimmune diseases. Gal-9^+^ Th cells expand the population by exogenously applied recombinant Gal-9, and the cells can be purified using Gal-9 expressed on the cellular surface. These findings provide useful techniques for obtaining a large number of Gal-9^+^ Th cells and will help facilitate the study of Gal-9^+^ Th cells.

## Materials and Methods

### Ethics Statement

Human PBMCs were obtained from healthy adult volunteers with the approval of the ethical committee at Kagawa University, Faculty of Medicine. Written informed consent was obtained from all participants. Mice used in this research received humane care in accordance with international guidelines and national law. The study protocol was approved by the Animal Care and Use Committee of Kagawa University.

### Isolation and Culture of Mouse CD4^+^ CD62L^+^ Naïve T cells

C57BL/6J mice were purchased from Charles River Laboratories Japan (Yokohama, Japan). Gal-9 knockout (Gal-9 KO) mice were obtained from GalPharma (Takamatsu, Japan). All animals were maintained under standard conditions with a 12-h day/night rhythm and with *ad libitum* access to food and water. CD4^+^ CD62L^+^ naïve T cells were isolated from splenocytes of 8–10 week-old mice using a CD4^+^CD62L^+^ T cell Isolation Kit (Miltenyi Biotec, Bergisch Gladbach, Germany) according to the manufacturer’s instructions. CD4^+^ CD62L^+^ purity was >94%. Expression and purification of recombinant human stable Gal-9 has been previously described [26]. The recombinant protein was >95% pure on SDS-PAGE with an endotoxin level of <0.001 endotoxin units/µg. Isolated naïve T cells were cultured in 96-well plates at 2 × 10^5^ cells/well in RPMI 1640 containing 10% heat-inactivated fetal bovine serum, penicillin G (10 IU/mL, Sigma-Aldrich, St. Louis, MO, USA), and streptomycin (100 µg/mL, Sigma-Aldrich) for 96 h. For stimulation under neutral conditions, cells were cultured in anti-CD3-coated plates (BD Biosciences, Franklin Lakes, NJ, USA) in the presence of anti-CD28 (2 µg/mL, BD Biosciences) and mouse IL-2 (5 ng/mL, R&D Systems, Minneapolis, MN, USA). For Th17 skewing, cells were cultured in human TGF-β1 (3 ng/mL, R&D Systems) and mouse IL-6 (20 ng/mL, R&D Systems) under neutral conditions. Treg skewing was conducted under the same culture conditions for Th17-skewing but IL-6 was omitted. For some experiments, human stable Gal-9 (30 nM), lactose (30 or 100 mM), sucrose (30 mM), anti-mouse IL-10 blocking antibody (10 µg/mL, BioLegend, San Diego, CA, USA), anti-mouse IL-10 receptor blocking antibody (10 µg/mL, BioLegend), anti-mouse TGF-β blocking antibody (10 µg/mL, Abcam, Cambridge, MA, USA), or mouse IL-10 (1, 3 or 10 ng/mL, R&D Systems) was included in the culture.

### Isolation of Gal-9^+^ Th cells and Co-culture with Th17-skewed T cells

Naïve CD4^+^ T cells were isolated from splenocytes as described above. Gal-9^+^ and Gal-9^−^ Th cells were sorted by positive or negative surface Gal-9 expression, respectively, using an anti-mouse Gal-9 antibody (clone 108A2, BioLegend) and a FACSAria cell sorter (BD Biosciences). Cell purity was >97%. For co-culture experiments, naïve T cells (5 × 10^4^) were cultured under Th17-skewing conditions for 6 h, and then co-cultured with Gal-9^+^ or Gal-9^−^ Th cells (5 × 10^4^) for an additional 90 h.

### ELISA

Quantification of mouse Gal-9 was carried out using ELISA as described previously with minor modifications [27]. Briefly, 96-well plates were coated with an anti-mouse Gal-9 antibody (Clone 108A2), blocked with 3% fetal bovine serum in phosphate-buffered saline, and then incubated with culture supernatant. Gal-9 was detected using polyclonal anti-mouse Gal-9 antibody conjugated with biotin (GalPharma) and streptavidin-conjugated horseradish peroxidase (Thermo Fisher Scientific, Waltham, MA, USA). After color development with tetramethyl benzidine (KPL, Gaithersburg, MD, USA), Gal-9 was quantified using a standard curve constructed with a recombinant mouse Gal-9. Mouse Gal-9 ELISA cannot be used to detect human stable Gal-9. Human Gal-9 ELISA was reported previously [27]. IL-17 was measured using a specific ELISA kit from R&D Systems according to the manufacturer’s instructions.

### Flow Cytometric Analysis

CD4 T cells were evaluated by flow cytometry using the following antibodies: anti-mouse CD3-PerCP (BD Biosciences), anti-mouse CD4-FITC (BD Biosciences or eBioscience, San Diego, CA, USA), anti-mouse Tim-3-PE (eBioscience), anti-mouse Gal-9-PE (clone 108A2, BioLegend) or biotinylated anti-mouse Gal-9 (clone 108A2, GalPharma), anti-mouse CD25-APC (BioLegend), anti-mouse IL-10-PE (BioLegend), anti-human Gal-9-Alexa488 (clone 9M1-3, GalPharma), anti-human CD3-PerCP (BD Biosciences), anti-human CD4-FITC (BioLegend), anti-human CD4-PE (BioLegend), anti-human CD25-APC (BioLegend), and anti-human/mouse Foxp3-PE (BioLegend). All data were acquired using a FACSCalibur cytometer (BD Biosciences) and analyzed with FlowJo software (Tree Star, Ashland, OR, USA).

### Isolation and Culture of Human Peripheral Blood CD4 T cells

PBMCs from healthy donors were prepared using a Lymphocyte Separation Kit (Nakalai, Kyoto, Japan). CD4 T cells were isolated using a CD4 T Cell Isolation Kit II (Miltenyi Biotec) according to the manufacturer’s instructions. The CD4 T cells (2 × 10^5^ cells/well) were cultured under TCR stimulation (anti-CD28 (2 µg/mL, BD Biosciences) and IL-2 (5 ng/mL, R&D Systems) in anti-CD3-coated plates) or left unstimulated for 96 h, and cell-surface Gal-9 expression or released Gal-9 in the culture media was measured. To determine cytokine mRNA expression, CD4 T cells cultured under TCR stimulation for 4 days were sorted into Gal-9^+^ CD25^+^ cells and Gal-9^−^ CD25^+^ cells using a FACSAria cell sorter at a purity of >97%. Sorted cells were further cultured for 4 days under TCR stimulation. Human Th17 cell development was performed as reported previously [28]. Briefly, CD4 T cells were cultured under TCR stimulation as described above in the presence or absence of IL-1β (50 ng/mL, R&D Systems), IL-6 (20 ng/mL, R&D Systems) and IL-23 (50 ng/mL, R&D Systems) for 9 days.

### Real-time RT-PCR

mRNA levels were evaluated using the SYBR Green I-based real-time RT-PCR with an ABI PRISM 7000 sequence detector (Applied Biosystems, Foster City, CA, USA) as previously described [3]. All gene primers were obtained from Takara Bio (Otsu, Japan). Glyceraldehyde-3-phosphate dehydrogenase (GAPDH) mRNA levels were used as an internal standard for calibration.

### Statistical Analysis

For statistical comparisons, non-parametric two-tailed Mann-Whitney U-tests and one- or two-way analysis of variance were used. All statistical analyses were performed with Prism 5 software (Graphpad Software). A *p*-value of <0.05 was considered significant.

## Supporting Information

Figure S1 **Specificity of anti-Gal-9 antibody.** (**A**) Schematic drawing of wild-type Gal-9 and stable Gal-9. Gal-9 consists of 2 carbohydrate-recognition domains (CRD) at the N- and C-termini, tethered by a linker peptide. Stable Gal-9 is a gene-engineered linker-less Gal-9, which retains biological activity of wild-type Gal-9. (**B**) Anti-mouse Gal-9 antibody 108A2 recognizes linker peptide of mouse Gal-9 and does not cross-react with stable Gal-9. The indicated proteins were coated in ELISA plates and detected using the 108A2 antibody. Mean ± SD (n = 3). (**C**) Mouse Gal-9 ELISA is constructed by 108A2 antibody as the coating antibody and polyclonal anti-mouse Gal-9 antibody as the detection antibody. The ELISA is highly specific to mouse Gal-9 and does not cross-react to human stable Gal-9 at 0.37 µg/mL. When mouse Gal-9 is quantified in the presence of human stable Gal-9, the samples were diluted accordingly. Mean values (n = 2).
(TIF)

Figure S2 **Elimination of exogenously added Gal-9 bound on the cell surface by 30 mM lactose.** (**A**) Naïve CD4 T cells were incubated with biotinylated human stable Gal-9 (30 nM) for 30 min on ice followed by incubation with lactose or sucrose (30 mM) for 30 min on ice. Human stable Gal-9 bound on the cells was stained with streptavidin- APC and analyzed using flow cytometry. (**B**) Naïve CD4 T cells were cultured under neutral conditions for 4 days to allow expansion of Gal-9^+^ CD25^+^ Th cells. The cells were incubated in the presence or absence of 100 mM lactose for 30 min on ice before staining of surface Gal-9 and analysis by flow cytometry.
(TIF)

Figure S3 **Sorting of Gal-9** ^+^ **and Gal-9^−^ Th cells.** Naïve CD4 T cells were sorted into Gal-9^+^ and Gal-9^−^ Th cells using a FACSAria. The purities of Gal-9^+^ and Gal-9^−^ Th cells were more than 97%.
(TIF)

Figure S4 **Regulation of human Th17/Treg development by Gal-9.** (**A and B**) CD4 T cells were isolated from human peripheral blood (4 healthy donors) by magnetic sorting and were cultured with or without 4 days of TCR stimulation in the presence or absence of 30 nM human stable Gal-9. CD25^+^ CD4 T cells (**A**) or CD25^+^ Foxp3^+^ CD4 T cells (**B**) were determined using flow cytometry. (**C**) Human CD4 T cells from 4 healthy donors were cultured under TCR stimulation in the presence of indicated cytokines and in the presence or absence of 30 nM human stable Gal-9 for 9 days before IL-17 secretion was measured by ELISA. Results are shown as the mean ± SEM of quadruplicate experiments. ***, p<0.001. Data representative of 2 experiments are shown.
(TIF)

Figure S5 **Gal-9^+^ Th cells in various organs in mice.** The representative dot plots showing the existence of Gal-9^+^ Th cells in the indicated organs of Table 1 are shown. Events in the gate are cell-surface Gal-9-positive populations.
(TIF)

## References

[1] Seki M, Sakata K, Oomizu S, Arikawa T, Sakata A, et al (2007) Beneficial effect of galectin 9 on rheumatoid arthritis by induction of apoptosis of synovial fibroblasts. Arthr Rheumat 56: 3968–3976. 18050192 10.1002/art.23076

[2] Wiener Z, Kohalmi B, Pocza P, Jeager J, Tolgyesi G, et al (2007) TIM-3 is expressed in melanoma cells and is upregulated in TGF-beta stimulated mast cells. J Invest Dermatol 127: 906–914. 17096021 10.1038/sj.jid.5700616

[3] Seki M, Oomizu S, Sakata KM, Sakata A, Arikawa T, et al (2008) Galectin-9 suppresses the generation of Th17, promotes the induction of regulatory T cells, and regulates experimental autoimmune arthritis. Clin. Immunol 127: 78–88. 10.1016/j.clim.2008.01.006 18282810

[4] Arikawa T, Saita N, Oomizu S, Ueno M, Matsukawa A, et al (2010) Galectin-9 expands immunosuppressive macrophages to ameliorate T-cell-mediated lung inflammation. Eur J immunol 40: 548–558. 19902429 10.1002/eji.200939886

[5] Dardalhon VA, Anderson C, Karman J, Apetoh L, Chandwaskar R, et al (2010) Tim-3/galectin-9 pathway: regulation of Th1 immunity through promotion of CD11b+Ly-6G+ myeloid cells. J Immunol 185: 1383–1392. 20574007 10.4049/jimmunol.0903275 PMC2925247

[6] Tsuboi Y, Abe H, Nakagawa R, Oomizu S, Watanabe K, et al (2007) Galectin-9 protects mice from the Shwartzman reaction by attracting prostaglandin E2-producing polymorphonuclear leukocytes. Clin Immunol 124: 221–233. 17560833 10.1016/j.clim.2007.04.015

[7] Kojima K, Arikawa T, Saita N, Goto E, Tsumura S, et al (2011) Galectin-9 attenuates acute lung injury by expanding CD14- plasmacytoid dendritic cell-like macrophages. Am J Respir Crit Care Med 184: 328–339. 21562126 10.1164/rccm.201010-1566OC

[8] Nobumoto A, Oomizu S, Arikawa T, Katoh S, Nagahara K, et al (2009) Galectin-9 expands unique macrophages exhibiting plasmacytoid dendritic cell-like phenotypes that activate NK cells in tumor-bearing mice. Clin Immunol 130: 322–330. 18974023 10.1016/j.clim.2008.09.014

[9] Kadowaki T, Arikawa T, Shinonaga R, Oomizu S, Inagawa H, et al (2012) Galectin-9 signaling prolongs survival in murine lung-cancer by inducing macrophages to differentiate into plasmacytoid dendritic cell-like macrophages. Clin Immunol 142: 296–307. 22177847 10.1016/j.clim.2011.11.006

[10] Zhu C, Anderson AC, Schubart A, Xiong H, Imitola J, et al (2005) The Tim-3 ligand galectin-9 negatively regulates T helper type 1 immunity. Nat Immunol 6: 1245–1252. 16286920 10.1038/ni1271

[11] Oomizu S, Arikawa T, Niki T, Kadowaki T, Ueno M, et al (2012) Galectin-9 suppresses Th17 cell development in an IL-2-dependent but Tim-3-independent manner. Clin Immunol 143: 51–58. 22341088 10.1016/j.clim.2012.01.004

[12] Mishra R, Grzybek M, Niki T, Hirashima M, Simons K (2010) Galectin-9 trafficking regulates apical-basal polarity in Madin-Darby canine kidney epithelial cells. Proc Natl Acad Sci USA 107: 17633–17638. 20861448 10.1073/pnas.1012424107 PMC2955135

[13] Matsuura A, Tsukada J, Mizobe T, Higashi T, Mouri F, et al (2009) Intracellular galectin-9 activates inflammatory cytokines in monocytes. Genes Cells 14: 511–521. 19335620 10.1111/j.1365-2443.2009.01287.x

[14] Chabot S, Kashio Y, Seki M, Shirato Y, Nakamura K, et al (2002) Regulation of galectin-9 expression and release in Jurkat T cell line cells. Glycobiology 12: 111–118. 11886844 10.1093/glycob/12.2.111

[15] Niki T, Tsutsui S, Hirose S, Aradono S, Sugimoto Y, et al (2009) Galectin-9 is a high affinity IgE-binding lectin with anti-allergic effect by blocking IgE-antigen complex formation. J Biol Chem 284: 32344–32352. 19776007 10.1074/jbc.M109.035196 PMC2781649

[16] Wang F, Wan L, Zhang C, Zheng X, Li J, et al (2009) Tim-3-Galectin-9 pathway involves the suppression induced by CD4+CD25+ regulatory T cells. Immunobiology 214: 342–349. 19362679 10.1016/j.imbio.2008.10.007

[17] Mills KH (2008) Induction, function and regulation of IL-17-producing T cells. Eur J Immunol 38: 2636–2649. 18958872 10.1002/eji.200838535

[18] Asseman C, Powrie F (1998) Interleukin 10 is a growth factor for a population of regulatory T cells. Gut 42: 157–158. 9536936 10.1136/gut.42.2.157 PMC1726994

[19] Levings MK, Gregori S, Tresoldi E, Cazzaniga S, Bonini C, et al (2005) Differentiation of Tr1 cells by immature dendritic cells requires IL-10 but not CD25+CD4+ Tr cells. Blood 105: 1162–1169. 15479730 10.1182/blood-2004-03-1211

[20] Levings MK, Sangregorio R, Galbiati F, Squadrone S, de Waal Malefyt R, et al (2001) IFN-alpha and IL-10 induce the differentiation of human type 1 T regulatory cells. J Immunol 166: 5530–5539. 11313392 10.4049/jimmunol.166.9.5530

[21] Anderson AC, Anderson DE, Bregoli L, Hastings WD, Kassam N, et al (2007) Promotion of tissue inflammation by the immune receptor Tim-3 expressed on innate immune cells. Science 318: 1141–1143. 18006747 10.1126/science.1148536

[22] Nagahara K, Arikawa T, Oomizu S, Kontani K, Nobumoto A, et al (2008) Galectin-9 increases Tim-3+ dendritic cells and CD8+ T cells and enhances antitumor immunity via galectin-9-Tim-3 interactions. J Immunol 181: 7660–7669. 19017954 10.4049/jimmunol.181.11.7660 PMC5886706

[23] Seelenmeyer C, Wegehingel S, Tews I, Kunzler M, Aebi M, et al (2005) Cell surface counter receptors are essential components of the unconventional export machinery of galectin-1. J Cell Biol 171: 373–381. 16247033 10.1083/jcb.200506026 PMC2171196

[24] Klibi J, Niki T, Riedel A, Pioche-Durieu C, Souquere S, et al (2009) Blood diffusion and Th1-suppressive effects of galectin-9-containing exosomes released by Epstein-Barr virus-infected nasopharyngeal carcinoma cells. Blood 113: 1957–1966. 19005181 10.1182/blood-2008-02-142596

[25] Korn T, Bettelli E, Oukka M, Kuchroo VK (2009) IL-17 and Th17 Cells. Ann Rev Immunol 27: 485–517. 19132915 10.1146/annurev.immunol.021908.132710

[26] Nishi N, Itoh A, Fujiyama A, Yoshida N, Araya S, et al (2005) Development of highly stable galectins: truncation of the linker peptide confers protease-resistance on tandem-repeat type galectins. FEBS letters 579: 2058–2064. 15811318 10.1016/j.febslet.2005.02.054

[27] Nobumoto A, Nagahara K, Oomizu S, Katoh S, Nishi N, et al (2008) Galectin-9 suppresses tumor metastasis by blocking adhesion to endothelium and extracellular matrices. Glycobiology 18: 735–744. 18579572 10.1093/glycob/cwn062

[28] Wilson NJ, Boniface K, Chan JR, McKenzie BS, Blumenschein WM, et al (2007) Development, cytokine profile and function of human interleukin 17-producing helper T cells. Nat immunol 8: 950–957. 17676044 10.1038/ni1497

