# Supplementary figures and images for: Cell Surface Galectin-9 Expressing Th Cells Regulate Th17 and Foxp3+ Treg Development by Galectin-9 Secretion
Source: PLoS One. 2012 Nov 7;7(11):e48574. doi: 10.1371/journal.pone.0048574 (PMC3492452; doi:10.1371/journal.pone.0048574)

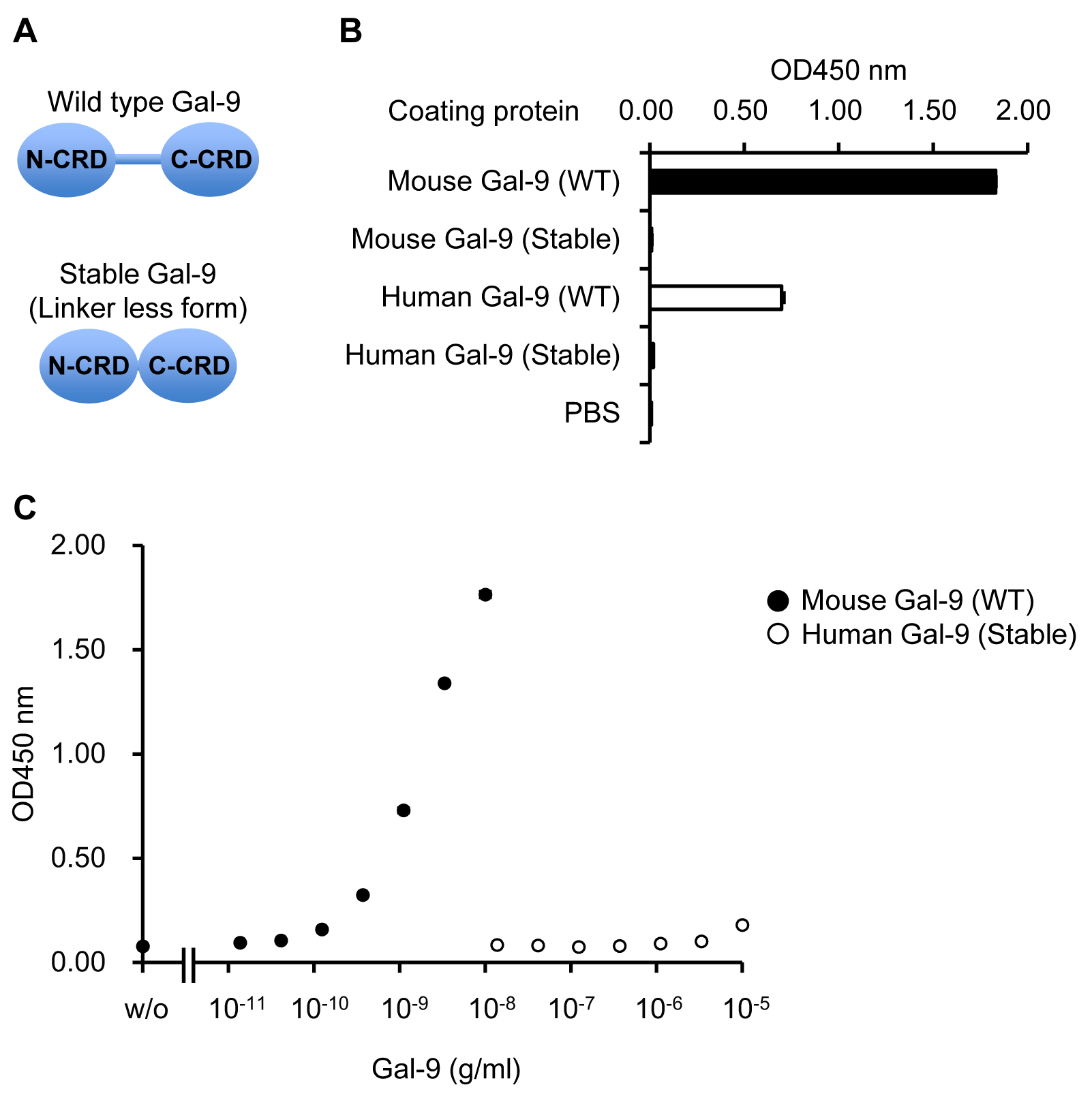

Supplement: Figure S1 — Specificity of anti-Gal-9 antibody. (A) Schematic drawing of wild-type Gal-9 and stable Gal-9. Gal-9 consists of 2 carbohydrate-recognition domains (CRD) at the N- and C-termini, tethered by a linker peptide. Stable Gal-9 is a gene-engineered linker-less Gal-9, which retains biological activity of wild-type Gal-9. (B) Anti-mouse Gal-9 antibody 108A2 recognizes linker peptide of mouse Gal-9 and does not cross-react with stable Gal-9. The indicated proteins were coated in ELISA plates and detected using the 108A2 antibody. Mean ± SD (n = 3). (C) Mouse Gal-9 ELISA is constructed by 108A2 antibody as the coating antibody and polyclonal anti-mouse Gal-9 antibody as the detection antibody. The ELISA is highly specific to mouse Gal-9 and does not cross-react to human stable Gal-9 at 0.37 µg/mL. When mouse Gal-9 is quantified in the presence of human stable Gal-9, the samples were diluted accordingly. Mean values (n = 2). (TIF) [file pone.0048574.s001.tif]

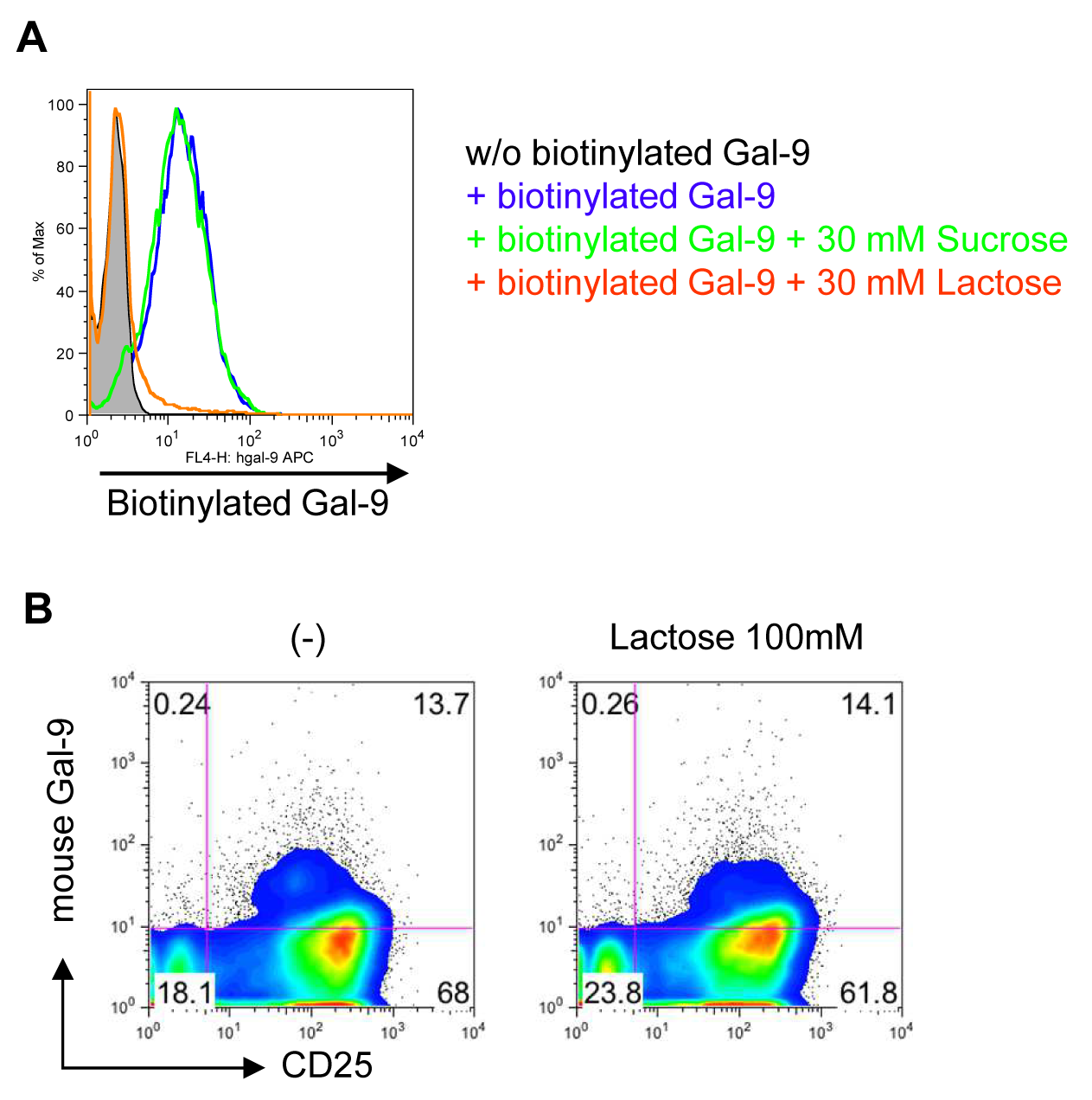

Supplement: Figure S2 — Elimination of exogenously added Gal-9 bound on the cell surface by 30 mM lactose. (A) Naïve CD4 T cells were incubated with biotinylated human stable Gal-9 (30 nM) for 30 min on ice followed by incubation with lactose or sucrose (30 mM) for 30 min on ice. Human stable Gal-9 bound on the cells was stained with streptavidin- APC and analyzed using flow cytometry. (B) Naïve CD4 T cells were cultured under neutral conditions for 4 days to allow expansion of Gal-9+ CD25+ Th cells. The cells were incubated in the presence or absence of 100 mM lactose for 30 min on ice before staining of surface Gal-9 and analysis by flow cytometry. (TIF) [file pone.0048574.s002.tif]

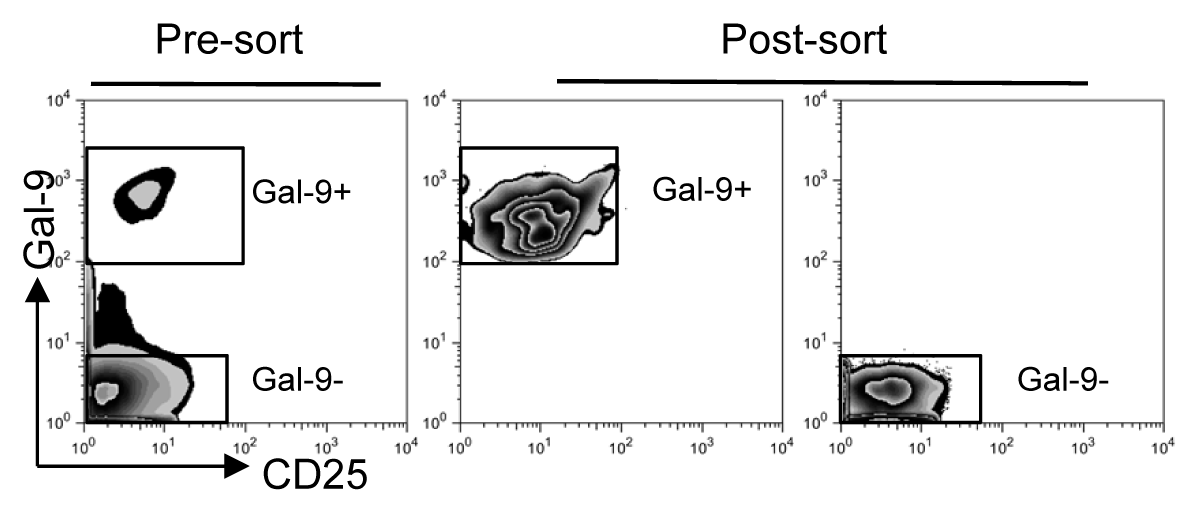

Supplement: Figure S3 — Sorting of Gal-9 + and Gal-9− Th cells. Naïve CD4 T cells were sorted into Gal-9+ and Gal-9− Th cells using a FACSAria. The purities of Gal-9+ and Gal-9− Th cells were more than 97%. (TIF) [file pone.0048574.s003.tif]

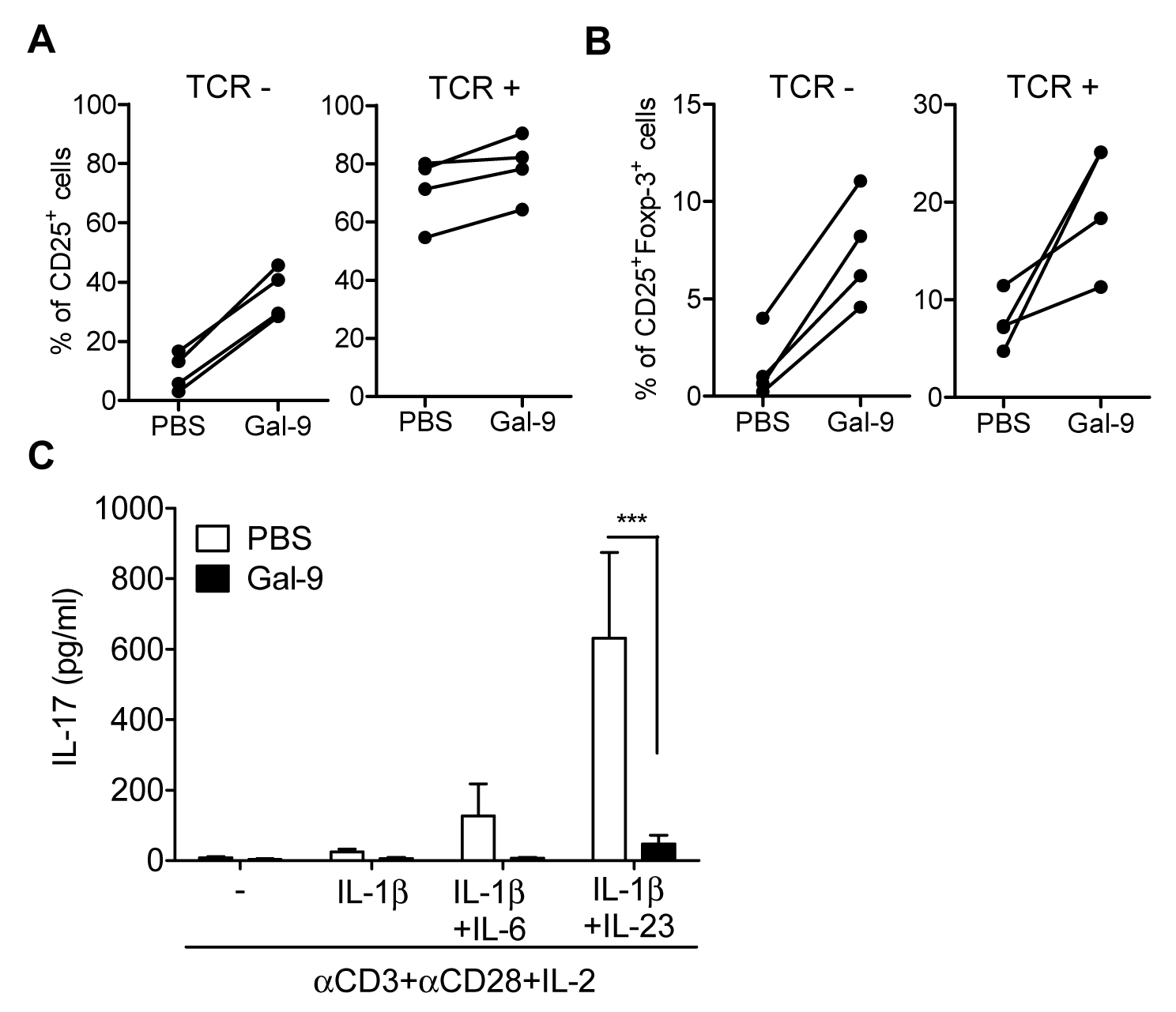

Supplement: Figure S4 — Regulation of human Th17/Treg development by Gal-9. (A and B) CD4 T cells were isolated from human peripheral blood (4 healthy donors) by magnetic sorting and were cultured with or without 4 days of TCR stimulation in the presence or absence of 30 nM human stable Gal-9. CD25+ CD4 T cells (A) or CD25+ Foxp3+ CD4 T cells (B) were determined using flow cytometry. (C) Human CD4 T cells from 4 healthy donors were cultured under TCR stimulation in the presence of indicated cytokines and in the presence or absence of 30 nM human stable Gal-9 for 9 days before IL-17 secretion was measured by ELISA. Results are shown as the mean ± SEM of quadruplicate experiments. ***, p<0.001. Data representative of 2 experiments are shown. (TIF) [file pone.0048574.s004.tif]

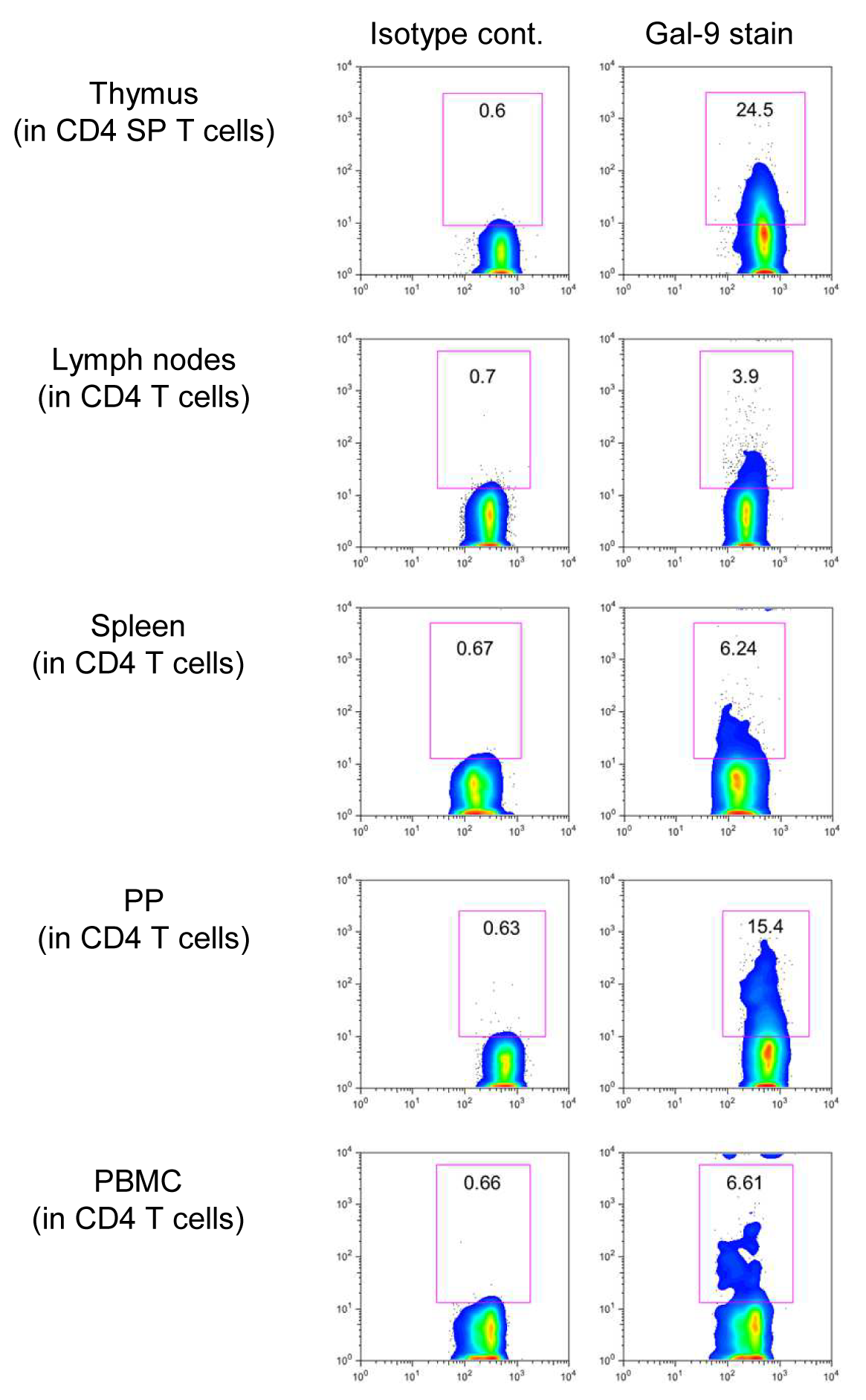

Supplement: Figure S5 — Gal-9+ Th cells in various organs in mice. The representative dot plots showing the existence of Gal-9+ Th cells in the indicated organs of Table 1 are shown. Events in the gate are cell-surface Gal-9-positive populations. (TIF) [file pone.0048574.s005.tif]
